# Population level effects of the active kids program on children and adolescents’ physical activity and sport participation in NSW, Australia

**DOI:** 10.1186/s12966-025-01763-2

**Published:** 2025-05-28

**Authors:** Katherine B. Owen, Bridget C. Foley, Lindsey J. Reece, William Bellew, Adrian Bauman

**Affiliations:** Prevention Research Collaboration, Sydney School of Public Health, The University of Sydney, Sydney, NSW Australia

**Keywords:** Children, Adolescents, Financial incentive, Voucher, Sport, Physical activity, Leisure-time, Evaluation, Policy, Behavior change

## Abstract

**Background:**

Active Kids was a universal program that aimed to reduce the cost of sport and active recreation programs for all school-enrolled children and adolescents (4.5–18 years) in New South Wales (NSW), Australia through provision of $100 vouchers. This study assesses trends in physical activity and sport participation in children and adolescents in NSW during its implementation to determine population level program effects.

**Methods:**

This study used the Active Kids program data from January 31 2018 to December 31 2022. Children and adolescents who registered in the program provided physical activity and sport participation data each year. The NSW Population Health Survey (PHS) and NSW AusPlay data measured the same outcomes from 2017 to 2022 in representative samples of the population. We calculated weighted prevalence estimates for physical activity and sport participation each year, and by age, gender, and socioeconomic status.

**Results:**

In the five-years of Active Kids program implementation, there was a slight decrease in the proportion of children and adolescents meeting physical activity guidelines (2018: 19.3%, 95% CI 19.2–19.4; 2022: 16.1%, 95% CI 16.0-16.2). In PHS between 2017 and 2022, there was also a slight decrease in the proportion of children and adolescents meeting physical activity guidelines (2017: 32.6%, 95% CI 29.1–36.0; 2022: 27.9%, 95% CI 24.4–31.5), with a larger drop in 2020 (24.4%, 95% CI 20.8–27.9). In the same period (2018–2022), there was a decrease in the proportion of children and adolescents who participated in sport at least once per week (2018: 70.3%, 95% CI 70.2–70.4; 2022: 53.6%, 95% CI 53.5–53.7). There was also a decrease in the proportion of children and adolescents who participated in sport at least once per week between 2017 and 2021 (2017: 78.2% 95% CI 74.5–81.9; 2021: 63.4%, 95% CI 60.1–66.8), with a slight increase in 2022 (69.8%, 95% CI 66.7–72.9).

**Conclusions:**

We found no increases in overall population levels of physical activity or sport participation among children and adolescents in NSW between 2017 and 2022. The single-component, universal program design should be modified, using targeted behaviour change theories, to address inequalities and stimulate population-level increases in physical activity and sport participation.

**Trial registration:**

Australian New Zealand Clinical Trials Registry (ACTRN12618000897268).

**Supplementary Information:**

The online version contains supplementary material available at 10.1186/s12966-025-01763-2.

## Background

Physical activity in children and adolescents is associated with improved physical, mental and cognitive health benefits [1]. The current Australian guidelines suggest that the benefits of physical activity are acquired through 60 min of moderate-to-vigorous physical activity (MVPA) each day [2]. However, it is estimated that 81% of adolescents worldwide and 82% of adolescents living in New South Wales (NSW) Australia do not meet these guidelines [3, 4].

The potential of sport and active recreation programs to boost physical activity participation levels is underutilised [5]. Children and adolescents who participate in sport have higher levels of physical activity and are more likely to remain active into adulthood [6]. However, only just over half of children (5–8 years: 57%; 9–14 years: 63%) and one third of adolescents (15–17 years: 68%) in Australia participate in sport at least once per week [7]. There is a need to increase population levels of sport participation during childhood and adolescence, both to increase physical activity, and to promote lifelong participation in health-enhancing behaviours.

One strategy increasingly used to drive population sport participation is the provision of financial incentives and sports voucher programs [8, 9]. These programs aim to reduce the costs to parents and caregivers, associated with sport participation (e.g., registration, equipment, uniforms) [10]. These previous programs have mostly targeted children [11–15], with few including adolescents [16]. Evidence for the effectiveness of financial incentives to increase physical activity or sport participation remains mixed ranging from studies reporting positive short and long-term effects [16], to others reporting only positive short-term effects [11–13], and to others still which report an absence of positive effects [14, 15, 17]. One financial incentive program in children, with population level evaluation, is the Child Fitness Tax Credit (CFTC) introduced in Canada in 2007 [17]. The program aimed to promote children’s fitness and offered parents tax credits to reduce the cost of registering their children in an approved physical activity program. The CFTC program ran for 10 years (2007–2016) and is estimated to have cost the government approximately $90 million to $115 million each year [18]. Nguyen and Grodendorst [17] conducted a population-level evaluation, using two large nationally representative surveys, and found that the CFTC had no effect on increasing overall physical activity participation for children in Canada. The evidence is thus equivocal overall, and currently insufficient to determine the effectiveness of financial incentive interventions in increasing children and adolescent’s physical activity and sport participation, especially at a population level.

In Australia, in 2018 the NSW Government Office of Sport implemented a $650 million universal voucher program, entitled Active Kids [19]. The voucher program aimed to increase sport participation by reducing the cost barrier. A specific long-term objective of the program was to increase population levels of physical activity in children and adolescents aged 5–17 years living in NSW. More than 1.2 million school enrolled children and adolescents were eligible to receive one or two AUD $100 vouchers per year. It is important to note that the Active Kids program began in 2018 and was implemented as intended for two years prior to the onset of the COVID-19 pandemic. Between March and June of 2020, NSW experienced community restrictions where sport and active recreation programs were mostly postponed, modified, or cancelled [20].

To assess the effectiveness of the Active Kids program, the NSW Government commissioned an independent pragmatic evaluation of the program. This evaluation has previously reported on parental awareness [21], population reach [22], and short-term effects [23] of the program. The program reached more than half of all eligible children and adolescents in NSW; however, those who spoke a primary language other than English at home, were aged 15–18 years old, lived in the most disadvantaged areas, and girls, were less likely to register compared to their counterparts [22]. Whilst these subgroups were less likely to register, the overall number of children in these subgroups who registered for a voucher increased over the program duration [24]. The children and adolescents who registered and used their voucher reported increasing their days achieving physical activity guidelines from four days per week to five days per week after six months [23]. This increased physical activity was observed across all subgroups. However, it is unclear whether increasing physical activity and sport participation among those who used a voucher is sufficient to influence population levels of physical activity for all children and adolescents in NSW.

Independent evaluations of large-scale public sector interventions are essential to inform policy and practice [25]. Given the high costs associated with population-wide interventions, it is important to understand whether these interventions are being used, are being implemented as intended, and are having the desired positive impact on health outcomes [26]. Evaluation findings can be used to adjust interventions or inform future interventions around the world to optimise health outcomes. Therefore, this study assessed trends in physical activity and sport participation among children and adolescents in NSW during the period of the Active Kids program to assess if population levels increased.

## Methods

### Study design

This study is a natural experiment using a repeated cross-sectional study design, and part of the Active Kids state-wide program evaluation [27].

### Active kids

The Active Kids program is a NSW-wide, whole-of-government initiative led by the NSW Government Office of Sport [19]. The Office of Sport led the development of and implementation of the Active Kids program, and the NSW Government department ‘Service NSW’ conducted the voucher administration and program provider approval through a centralised government platform. There was no explicit theoretical framework which underpinned the design of the intervention. The program offered all school-aged children and adolescents between 4.5 and 18 years a financial voucher (AUD $100) to reduce the cost of registration in an approved structured physical activity or sport program. In 2018, one voucher per child was available, and this increased to two vouchers per child, each year from 2019 to 2022. For activities to be eligible, they needed to: (1) be part of an organised program of at least 8 weeks; and (2) include moderate or vigorous levels of physical activity. Examples of eligible activities include team sports such as basketball or soccer, individual sports such as tennis, and active recreation such as scouts. Sporting clubs or associations and active recreation providers that met the eligibility criteria could register to be an Active Kids provider through the Service NSW website.

### Active kids registration data

The Active Kids program was administered through a bespoke government platform, where the parent/carer was required to register the child and complete mandatory questions regarding the child’s demographics, physical activity, and sport participation. The specific measures used are described after the data sources have been presented. The Active Kids physical activity and sports questions asked at registration were consistent with concurrent data collected through the NSW statewide Population Health (PHS) and Sport Australia’s AusPlay survey, respectively.

### NSW population health survey (PHS) (2017–2022)

The NSW PHS is an ongoing health surveillance study conducted by the NSW Government, administered using computer assisted telephone interviews (CATI). The survey utilises a dual-frame design to include both mobile phone and landline users that have been randomly sampled [28]. The survey data is weighted to account for the probability of selection through the landline and mobile frames and to match Australian Bureau of Statistics Local Health District, gender, and age estimates [29]. In 2017, the response rate was 24% and decreased over time to 9% in 2022.

### AusPlay (2017–2022)

Ausplay is a national population sport participation survey conducted by the Australian Sports Commission (Sport Australia) using CATI. Between 2017 and June 2019, the survey used an overlapping dual frame design including both mobile phone and landline users. In July 2019, AusPlay shifted to a single frame, using one sample source, a random sample of mobile phone numbers. The survey data is weighted to account for the probability of selection through the landline and mobile frames (accounting for the change to a single mobile frame) and to match Australian Bureau of Statistics Local Health District, gender, and age estimates. During the time period, the response rate remained relatively consistent at approximately 13% [30]. We limited the Ausplay data to NSW.

### Measures

#### Physical activity

Meeting physical activity guidelines was assessed in the Active Kids registration platform and in the NSW PHS. In the Active Kids registration platform, the parent/carer was asked: “In a typical week, how many days was the child physically active for at least 60 minutes? This could be made up of different activities including walking, cycling to school, and sport at lunchtime or an exercise class” [31]. Responses ranged from 0 to 7 days and children and adolescents were classified as meeting current Australian physical activity guidelines if they were active for at least 60 min on 7 days. This proxy self-report single item demonstrated to be a reliable and valid assessment of youth physical activity [32]. In the NSW PHS, the parent/carer was asked: “On about how many days during the school week does child usually do physical activity outside of school hours?”, “On those days, about how many hours does child usually do physical activity?”, “On about how many weekend days does child usually do physical activity?” “On a typical weekend day, about how many hours does child usually do physical activity?”. Meeting physical activity guidelines was defined as at least one hour or more of moderate or vigorous physical activity outside of school hours each day.

#### Sport participation

Sport participation was measured in the Active Kids registration platform and in Ausplay. The parent/carer was asked: “Approximately, how many organized sessions of sport or physical activities has the child participated in, outside of school hours, during the last 12 months?” The parent/carer had the option to respond by entering the number of times in the last 12 months, number of times per month, or number of times per week. Children and adolescents were classified as participating in sport at least once per week if they participated in at least 52 sessions per year.

#### Demographic characteristics

Demographic characteristics included date of birth (age), sex, and postcode (area-level socioeconomic status). Area-level socioeconomic status was categorised using postcode of residence according to the Australian Bureau of Statistic’s Socio-Economic Index For Areas (SEIFA) Index of Relative Disadvantage [33]. In the NSW PHS, the quintiles were pre-classified into five categories, whereas in the Active Kids data and AusPlay we used quartiles for consistency with previous work.

### Data analysis

Descriptive statistics, including frequencies and proportions, were calculated for demographic characteristics of children and adolescents who registered in the Active Kids program each year. These were compared against the National 2021 census conducted by the Australian Bureau of Statistics. Due to the large sample size, proportional reporting ratios (PRR) were calculated to quantify the magnitude of differences between children and adolescents registered in the program and all eligible children and adolescents [34]. We calculated the average PRR from 2017 to 2022. Weighted prevalence estimates and 95% confidence intervals (CIs) were calculated for physical activity and sport participation each year, and by age, gender, and socioeconomic status. To examine trends, multivariable logistic regression models were used, including interaction terms for year and the demographic characteristic. Linearity of the trend was tested by entering year as both a continuous and a categorical variable and performing a likelihood ratio test to compare the two models. We also examined whether the gap in regular sport participation between age groups, sexes, and socioeconomic status groups changed over time using multivariable log-Poisson regression models, presented as prevalence ratios (PR) with 95% CIs. All analyses were conducted in SAS Version 9.4.

## Results

Between 2018 and 2021 the number of children and adolescents in NSW who registered for an Active Kids voucher increased to from 664,973 to 855,597, with a decrease in 2022 (*n* = 766,089) (Table 1). In comparison to the overall eligible population of children and adolescents, this represents 44% in 2018, 57% in 2021 and 51% in 2022. The reach of the program was greater among younger children (4–11 years), males, and children and adolescents living in the least disadvantaged areas (4th SEIFA quartile).

**Table 1:** Demographic characteristics of children registering in the active kids program by year

	2018		2019		2020		2021		2022		All eligible children in NSW in 2021		aPRR
	*N*	%	*N*	%	*N*	%	*N*	%	*N*	%	*N*	%	
All persons	664,973	100.0	777,240	100.0	795,528	100.0	855,597	100.0	766,089	100.0	1,508,992	100.0	
Age category													
4–8 years	267,247	40.2	319,445	41.1	321,274	40.4	346,350	40.5	309,876	40.5	494,916	33.7	1.20
9–11 years	183,855	27.7	208,548	26.8	208,242	26.2	218,423	25.5	187,476	24.5	303,868	20.7	1.26
12–14 years	136,686	20.6	158,177	20.4	166,421	20.9	177,879	20.8	158,198	20.7	299,260	20.4	1.01
15–18 years	77,185	11.6	91,070	11.7	99,591	12.5	112,945	13.2	110,539	14.4	368,933	25.1	0.50
Sex													
Male	358,223	53.9	408,998	52.6	416,606	52.4	446,356	52.2	399,675	52.2	754,496	50.0	1.06
Female	305,778	46.0	366,921	47.2	376,786	47.4	406,593	47.5	361,847	47.2	754,496	50.0	0.94
Missing/Prefer not to say	972	0.2	1,321	0.2	2,136	0.3	2,648	0.3	4,567	0.6			
Socio-economic status													
1st quartile (most disadvantaged)	107,505	16.2	128,146	16.5	126,847	16.0	142,142	16.6	132,971	17.4	348,619	23.8	0.70
2nd quartile	151,501	22.8	176,895	22.8	176,368	22.2	190,975	22.3	171,970	22.5	367,545	25.1	0.90
3rd quartile	171,687	25.8	207,534	26.7	214,292	26.9	231,272	27.0	209,467	27.3	306,329	20.9	1.29
4th quartile (least disadvantaged)	217,978	32.8	264,044	34.0	277,819	34.9	290,966	34.0	251,363	32.8	442,902	30.2	1.12
Missing	16,302	2.5	621	0.1	202	0.0	242	0.0	318	0.0			

Note. aPRR = average proportional reporting ratios

### Physical activity

**Fig. 1:**
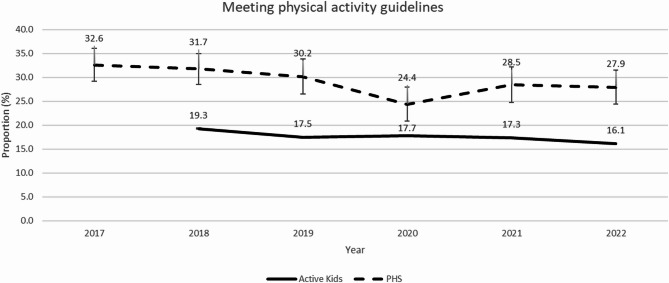
The proportion of children meeting physical activity guidelines in the Active Kids program data and NSW PHS by year

Active Kids program registration data revealed a slight decrease in the proportion of children and adolescents meeting physical activity guidelines each year when they registered in the Active Kids program (19% in 2018 to 16% in 2022) (Fig. 1). In the independently collected PHS data between 2017 and 2022, there was also a slight decrease in the proportion of children and adolescents meeting physical activity guidelines (33% in 2017 to 28% in 2022), with a larger drop in 2020 (24%) (Fig. 1). Trend analyses indicated that these decreasing trends were non-linear in both datasets (*p* < 0.001).

In the Active Kids program, there was a decrease in children and adolescents of all age groups meeting physical activity guidelines, however this decrease was steepest in the 9–11-year-old age group (20–15%; p for interaction < 0.001; Table 2). There was a decrease in both males and females meeting physical activity guidelines, with a steeper decrease in males (23–18%; p for interaction < 0.001). Children and adolescents across all socioeconomic quartiles showed similar decreases in meeting physical activity guidelines (p for interaction < 0.001).

**Table 2:** The proportion of children aged 4–18 meeting physical activity guidelines by year in NSW (Active Kids)

	2018 (*n* = 664973) % (95% CI)	2019 (*n* = 777240) % (95% CI)	2020 (*n* = 795528) % (95% CI)	2021 (*n* = 736245) % (95% CI)	2022 (*n* = 766089) % (95% CI)
All children	19.27 (19.17, 19.36)	17.45 (17.37, 17.54)	17.74 (17.66, 17.83)	17.34 (17.25, 17.42)	16.09 (16.01, 16.17)
Age group					
4–8 years	22.76 (22.6, 22.92)	21.54 (21.4, 21.69)	22.62 (22.47, 22.76)	22.11 (21.96, 22.26)	20.93 (20.79, 21.08)
9–11 years	19.56 (19.38, 19.75)	17.22 (17.06, 17.38)	17.43 (17.27, 17.59)	16.97 (16.81, 17.14)	15.2 (15.04, 15.36)
12–14 years	15.37 (15.18, 15.56)	12.85 (12.69, 13.02)	12.85 (12.69, 13.01)	12.73 (12.57, 12.9)	11.66 (11.5, 11.82)
15–18 years	13.38 (13.14, 13.62)	11.61 (11.4, 11.82)	10.85 (10.65, 11.04)	11.03 (10.83, 11.23)	10.36 (10.18, 10.54)
Gender					
Males	22.66 (22.52, 22.79)	20.17 (20.05, 20.29)	20.55 (20.43, 20.68)	20.07 (19.94, 20.19)	18.4 (18.28, 18.52)
Females	15.32 (15.19, 15.45)	14.42 (14.31, 14.54)	14.66 (14.54, 14.77)	14.33 (14.21, 14.44)	13.57 (13.46, 13.68)
Socioeconomic status					
1st (most disadvantaged)	17.88 (17.66, 18.11)	15.6 (15.4, 15.8)	15.42 (15.23, 15.62)	15.07 (14.86, 15.27)	13.91 (13.73, 14.1)
2nd	20.47 (20.27, 20.67)	18.44 (18.26, 18.62)	18.96 (18.78, 19.15)	18.38 (18.2, 18.57)	17.66 (17.48, 17.84)
3rd	18.72 (18.54, 18.91)	17.04 (16.88, 17.2)	17.39 (17.23, 17.55)	17.05 (16.89, 17.22)	15.92 (15.76, 16.08)
4th (least disadvantaged)	19.69 (19.52, 19.86)	18.01 (17.86, 18.16)	18.3 (18.16, 18.44)	17.93 (17.78, 18.08)	16.31 (16.16, 16.45)

In the NSW PHS, there was a decrease in children and adolescents of all age groups meeting physical activity guidelines (Table 3), with the steepest decline in the 9–11-year-old age group (26–17%) followed by the 15–18-year-old age group (58 to 53%; p for interaction = 0.13). There was a decrease in both males and females meeting physical activity guidelines, with a steeper decrease in males (38–33%; p for interaction = 0.08). Children and adolescents across all socioeconomic status categories reported similar decreases in the proportion of children and adolescents meeting physical activity guidelines (p for interaction = 0.76). In the NSW PHS there was no evidence that the gap in meeting physical activity guidelines between age groups, sexes, and socioeconomic status groups changed between 2017 and 2022 (Supplementary Table 1).

**Table 3:** The proportion of children aged 5–18 meeting physical activity guidelines in NSW (PHS)

	2017 (*n* = 1655) % (95% CI)	2018 (*n* = 1657) % (95% CI)	2019 (*n* = 1467) % (95% CI)	2020 (*n* = 1617) % (95% CI)	2021 (*n* = 1490) % (95% CI)	2022 (*n* = 1445) % (95% CI)
All children	32.58 (29.14, 36.02)	31.74 (28.52, 34.95)	30.16 (26.53, 33.79)	24.35 (20.8, 27.9)	28.46 (24.78, 32.15)	27.94 (24.42, 31.47)
Age group						
5–8 years	28.85 (22.1, 35.61)	28.45 (22.33, 34.57)	28.05 (21.13, 34.96)	19.35 (13.4, 25.29)	23.61 (17.39, 29.83)	29.19 (22.43, 35.95)
9–11 years	25.65 (18.81, 32.5)	25.73 (19.27, 32.18)	24.2 (16.81, 31.6)	11.76 (7.1, 16.42)	22.94 (15.46, 30.41)	16.47 (10.62, 22.32)
12–14 years	18.14 (12.66, 23.63)	18.72 (13.72, 23.73)	13.64 (9.36, 17.93)	10.86 (6.22, 15.5)	16.84 (10.48, 23.19)	14.68 (9.67, 19.69)
15–18 years	57.51 (50.9, 64.11)	53.15 (46.29, 60)	54.03 (46.42, 61.65)	57.37 (49.4, 65.33)	51.63 (43.79, 59.47)	52.78 (44.5, 61.05)
Gender						
Males	38.48 (33.71, 43.25)	36.88 (32.28, 41.49)	32.22 (27.46, 36.99)	26.94 (22.14, 31.75)	29.96 (24.91, 35)	32.84 (27.82, 37.87)
Females	26.38 (21.47, 31.3)	26.25 (21.9, 30.6)	27.78 (22.21, 33.36)	21.67 (16.4, 26.95)	26.86 (21.45, 32.28)	21.9 (17.17, 26.64)
Socioeconomic status						
1st (most disadvantaged)	31.73 (24.07, 39.39)	30.78 (23.91, 37.65)	25.71 (18.59, 32.83)	24.7 (17.92, 31.47)	29.03 (20.79, 37.27)	22.5 (15.75, 29.25)
2nd	29.00 (22.15, 35.85)	30.17 (23.13, 37.21)	29.77 (21.89, 37.65)	25.26 (17.54, 32.97)	26.22 (18.89, 33.54)	29.44 (22.09, 36.79)
3rd	33.65 (26.63, 40.68)	29.42 (22.6, 36.25)	31.51 (23.38, 39.65)	27.24 (19.3, 35.18)	28.73 (21.66, 35.8)	31.65 (24, 39.3)
4th	39.62 (31.81, 47.42)	31.5 (24.81, 38.19)	30.26 (23.19, 37.33)	20.68 (13.19, 28.17)	32.2 (24.3, 40.1)	32.2 (23.12, 41.27)
5th (least disadvantaged)	29.1 (19.81, 38.39)	37.92 (29.47, 46.37)	36.21 (25.49, 46.92)	21.94 (11.78, 32.1)	26.71 (15.65, 37.78)	25.46 (16.38, 34.53)

Note. in the NSW PHS different physical activity questions are asked and meeting guidelines is classified differently for the 5–15-year age group and the 16–18-year age group. For children aged 5–15, meeting physical activity guidelines is defined as at least 60 min of physical activity each day; for children aged 16–18, the physical activity guidelines are classified as at least 150 min of physical activity a week

### Sport participation

**Fig. 2:**
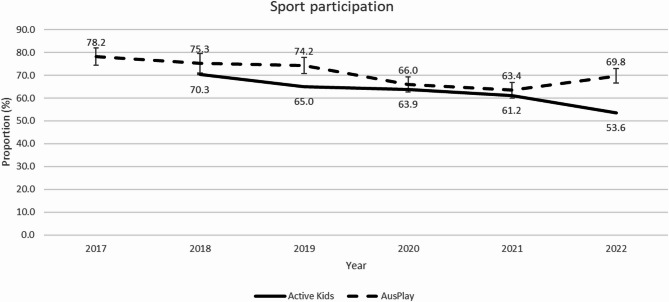
The proportion of children participating in sport at least once per week in the Active Kids program data and NSW AusPlay by year

In the Active Kids program between 2018 and 2022, there was a decrease in the proportion of children and adolescents who participated in sport at least once per week (70–54%; Fig. 2). In the NSW Ausplay, between 2017 and 2021, there was also a decrease in the proportion of children and adolescents who participated in sport at least once per week (78–63%), with a slight increase in 2022 (70%). Trend analyses indicated that these decreasing trends were non-linear in both datasets (*p* < 0.001).

In the Active Kids program, there was a decrease in the proportion of children and adolescents who participated in sport at least once per week for all age groups (Table 4), with the greatest decrease reported by 12–14 years (75–57%; p for interaction < 0.001). Among both males and females there was a decrease in sport participation, but there was a greater decrease for females (70–52%; p for interaction < 0.001). The decrease in sport participation was similar across all children and adolescents across all socioeconomic quartiles (p for interaction < 0.001).

**Table 4:** The proportion of children aged 4–18 years participating in sport at least once per week in NSW (Active Kids)

	2018 (*n* = 664973) % (95% CI)	2019 (*n* = 777240) % (95% CI)	2020 (*n* = 795528) % (95% CI)	2021 (*n* = 736245) % (95% CI)	2022 (*n* = 766089) % (95% CI)
All children	70.28 (70.17, 70.39)	65.01 (64.91, 65.12)	63.9 (63.8, 64.01)	61.19 (61.08, 61.3)	53.6 (53.50, 53.72)
Age group					
4–8 years	65.02 (64.84, 65.21)	60.24 (60.07, 60.41)	58.8 (58.63, 58.97)	56.06 (55.88, 56.24)	49.29 (49.11, 49.46)
9–11 years	72.86 (72.66, 73.07)	67.04 (66.84, 67.24)	66.52 (66.32, 66.72)	63.41 (63.2, 63.63)	55.44 (55.22, 55.67)
12–14 years	74.6 (74.36, 74.83)	68.96 (68.73, 69.19)	68.01 (67.78, 68.23)	65.2 (64.96, 65.43)	56.92 (56.67, 57.16)
15–18 years	74.64 (74.33, 74.95)	70.26 (69.96, 70.55)	68.04 (67.75, 68.33)	65.73 (65.43, 66.03)	57.88 (57.59, 58.17)
Gender					
Males	70.82 (70.67, 70.97)	66.15 (66, 66.29)	65.04 (64.89, 65.18)	62.35 (62.2, 62.51)	55.05 (54.9, 55.21)
Females	69.7 (69.54, 69.86)	63.81 (63.66, 63.97)	62.73 (62.57, 62.88)	59.97 (59.81, 60.13)	52.14 (51.98, 52.31)
Socioeconomic status					
1st (most disadvantaged)	59.36 (59.07, 59.66)	54.82 (54.55, 55.09)	53.01 (52.74, 53.28)	49.28 (48.99, 49.56)	42.45 (42.18, 42.71)
2nd	68.13 (67.89, 68.36)	63.12 (62.89, 63.34)	61.87 (61.65, 62.1)	57.91 (57.67, 58.15)	51.76 (51.53, 52)
3rd	70.96 (70.74, 71.17)	65.6 (65.4, 65.8)	64.18 (63.98, 64.38)	61.35 (61.13, 61.56)	53.68 (53.47, 53.9)
4th (least disadvantaged)	76.99 (76.81, 77.17)	70.77 (70.6, 70.95)	69.96 (69.79, 70.13)	68.76 (68.58, 68.95)	60.72 (60.53, 60.91)

In the NSW Ausplay, there was a decrease in the proportion of children and adolescents who participated in sport at least once per week for all age groups, with the steepest decrease reported by 4–8 years (73–49% in 2021, and 57% in 2022; p for interaction = 0.75; Table 5). The decrease in sport participation was greater in males (79–63% in 2021, and 69% in 2022; p for interaction = 0.049). Children and adolescents living in the most disadvantaged areas reported the greatest decrease in sport participation (71–57% in 2021, and 58% in 2022; p for interaction = 0.91). However, in the NSW Ausplay there was no evidence that the gap in sport participation between age groups, sexes, and socioeconomic status groups changed over the study period (Supplementary Table 2).

**Table 5:** The proportion of children aged 4–18 years participating in sport at least once per week in NSW (Ausplay)

	2017 (*n* = 805) % (95% CI)	2018 (*n* = 798) % (95% CI)	2019 (*n* = 992) % (95% CI)	2020 (*n* = 1231) % (95% CI)	2021 (*n* = 1268) % (95% CI)	2022 (*n* = 1180) % (95% CI)
All children	78.16 (74.46, 81.87)	75.25 (70.99, 79.51)	74.22 (70.66, 77.79)	65.96 (62.7, 69.22)	63.44 (60.11, 66.77)	69.76 (66.65, 72.87)
Age group						
4–8 years	73.09 (66.29, 79.89)	66.43 (58.7, 74.15)	63.52 (56.74, 70.3)	52.12 (46.31, 57.93)	49.16 (42.82, 55.5)	57.31 (51.56, 63.06)
9–11 years	77.79 (70.48, 85.1)	84.2 (78.23, 90.17)	78.45 (71.7, 85.2)	72.00 (65.45, 78.56)	64.9 (58.66, 71.13)	71.81 (65.28, 78.34)
12–14 years	78.97 (71.7, 86.24)	76.05 (69.19, 82.91)	74.19 (67.62, 80.76)	65.88 (59.3, 72.46)	64.89 (59.27, 70.52)	70.65 (65.08, 76.22)
15–18 years	89.2 (82.52, 95.87)	88.33 (81.96, 94.69)	92.17 (87.84, 96.5)	86.93 (81.84, 92.02)	85.59 (80.48, 90.7)	91.05 (86.64, 95.45)
Gender						
Males	78.56 (73.6, 83.53)	71.51 (66.18, 76.83)	73.08 (68, 78.16)	64.8 (60.22, 69.38)	62.96 (58.25, 67.68)	69.39 (65.22, 73.56)
Females	77.69 (72.14, 83.25)	79.5 (72.65, 86.36)	75.59 (70.67, 80.5)	67.16 (62.55, 71.78)	63.93 (59.22, 68.63)	70.15 (65.5, 74.8)
Socioeconomic status						
1st (most disadvantaged)	71.05 (60.52, 81.58)	60.92 (45.8, 76.03)	68.54 (58.4, 78.69)	59.04 (50.44, 67.64)	56.98 (47.64, 66.31)	57.96 (48.67, 67.26)
2nd	79.55 (71.26, 87.85)	75.32 (66.82, 83.83)	65.08 (56.52, 73.64)	61.51 (54.61, 68.4)	62.34 (54.57, 70.11)	64.33 (57.02, 71.64)
3rd	76.11 (68.67, 83.54)	76.59 (69.45, 83.73)	76.73 (69.95, 83.51)	61.71 (54.8, 68.62)	64.86 (58.51, 71.2)	69.86 (63.73, 75.98)
4th (least disadvantaged)	82.72 (77.21, 88.24)	80.33 (74.44, 86.21)	79.74 (74.46, 85.03)	75.32 (70.32, 80.32)	66.73 (61.7, 71.76)	76.71 (72.16, 81.26)

## Discussion

This study assessed population level trends in physical activity and sport participation among children and adolescents in NSW during the period of the Active Kids program. Active Kids program data indicated that there was a slight decrease in the proportion of children and adolescents who met physical activity guidelines and participated in sport once per week between 2018 and 2022. Population data confirmed that there was no evidence of an increase in physical activity or sport participation for children and adolescents in NSW between 2017 and 2022. Specifically, the proportion of children and adolescents meeting physical activity guidelines decreased slightly between 2017 and 2022, with a larger drop during the initial COVID-19 period in 2020. The proportion of children and adolescents regularly playing sport also decreased slightly between 2017 and 2021, with a slight increase in 2022. The results of the Active Kids evaluation need to be interpreted with COVID-19 in mind, and it is possible that the program may have attenuated the impact of the COVID-19 community restrictions on sport participation in NSW.

For the large number of children and adolescents who registered and used their Active Kids voucher, there were increases in physical activity levels, which persisted six-months after using a voucher [23]. The program had very high population reach, with 53% of all eligible children and adolescents in NSW registering and 43% of eligible children and adolescents using their voucher. However, despite these positive results for children and adolescents who engaged in the program which suggest strong internal validity of the program, the evidence for the external validity is less robust. There were no increases in physical activity or sport participation at the population level, indicating that while the program effectively increased activity among participants, it was not generalizable enough to impact overall levels of physical activity or sport participation across the population. These findings suggest that voucher programs should be part of a comprehensive systems-approach to increase population levels of physical activity [5]. When combined with other coordinated public health programs such as improved walking and cycling infrastructure and mass media strategies, voucher programs may influence population levels of physical activity and sport participation [35].

These findings are consistent with the population level evaluation of the Canadian CFTC. The CFTC ran for 10 years with substantial costs for government [18], and had no effect on increasing physical activity participation for children in Canada [17]. Given the substantial costs and limited evidence for the effectiveness of financial incentive programs to increase physical activity or sport participation at the population level, these universal and single-component programs should be reconsidered. Future voucher programs could consider proportionate universal approaches that strategically allocate program funds to less active populations for whom cost is a major barrier to participation (e.g., mass media campaigns and partnerships with community leaders and organisations to encourage further uptake in targeted children and adolescents). In 2024, the Active Kids program was adapted to be means-tested, so that only families who are eligible for the Family Tax Benefit were eligible for the vouchers. This change may contribute to closing the socioeconomic gap in physical activity and this adapted program should be evaluated.

Evaluation of large-scale policies and programs should always consider effects on existing inequalities. Using population level data, this study found that in NSW, between 2017 and 2022 children and adolescents from disadvantaged backgrounds were less active, and this socioeconomic gap remained consistent for physical activity and sport. Our study also found that, females reported lower levels of physical activity and sport than males and the gender gap did not widen. This finding is important given concerns that universal interventions, like the Active Kids program, might widen existing inequalities by disproportionately benefiting already active or advantaged groups [36]. Similarly, previous Active Kids evaluation findings demonstrated that children and adolescents who were female or from disadvantaged backgrounds initially had lower levels of physical activity and were less likely to engage in the program. However, the children and adolescents from these less active subgroups who registered and used a voucher reported similar increases in physical activity and sport participation, with disparities most significantly reduced within 8 weeks of using a voucher [23]. This suggest that additional vouchers, or complementary intervention components (e.g. female only activities, support for businesses to deliver programs in disadvantaged areas, transport or meal provision for children and adolescents) may be required for these less active subgroups to sustain the reductions in inequalities achieved in the first 8 weeks after using a voucher. Behaviour change theories should be utilised to integrate the most appropriate intervention components or modify existing components (e.g. fiscal value, frequency of vouchers). The Global Action Plan on Physical Activity 2018–2030 [5] prioritises reducing inequalities and suggests that to see progress requires a coordinated and strategic systems approach which focuses on three areas of action (i) innovative and diverse financing mechanisms; (ii) coherent policy, laws, regulatory frameworks, and standards; and (iii) more integrated delivery of physical activity [37]. A specific targeted focus on addressing the inequalities in physical activity and sport participation amongst children and adolescents must remain a priority to achieve population level behaviour change.

The findings of the Active Kids evaluation need to be interpreted with COVID-19 in mind. The Active Kids program began in 2018 and was implemented as intended for two years prior to the 2020 onset of the COVID‐19 pandemic. However, even prior to COVID-19, there was a slight decrease in sport participation for children and adolescents in NSW (2017 to 2019). During the NSW COVID‐19 community restrictions, parents/carers of children and adolescents in the Active Kids program reported that 40% of children and adolescent’s voucher activities were postponed, 38% were continuing but in a modified form, 12% were cancelled and only 6% remained unaffected [20]. Our study found that physical activity and sport participation declined in 2020 for all socioeconomic groups, ages and genders and is slowly returning to pre- COVID‐19 levels in NSW. In Western Australia, where there were minimal COVID-19 related community restrictions, the decrease in children and adolescent’s sport participation was slightly steeper than in NSW, dropping from 74% in 2017 to 56% in 2021, with an increase in 2022 (61%) [38]. This suggests that the Active Kids program may have attenuated the impact of the COVID-19 community restrictions on sport participation in NSW and may have aided the recovery and survival of sport in NSW.

This study was among the first to triangulate multiple representative population-level datasets to evaluate a large scale universal recreational physical activity and organised sport voucher program over multiple years. Using these datasets provides external validity to our evaluation findings, and provides policy makers, academics, and practitioners, with robust data to make judgments on the population impact of the Active Kids program. The use of consistent and validated survey questions within the Active Kids program evaluation allowed comparisons across population datasets. Alongside providing evaluation data for the Active Kids program, this study provides trend data on physical activity and sport participation in children and adolescents in NSW across a six-year period. Limitations of this study included only having self-reported measures for physical activity and sport participation, however this is common practice in population health surveillance. All physical activity and sport participation questions were asked of the child or adolescents’ parent/carer by proxy, potentially introducing social desirability bias and recall bias [39]. Additionally, while self-report the single item physical activity question used in the Active Kids registration platform has been demonstrated to be reliable and valid in assessing physical activity [32], it is not recommended for assessing within-individual change over time [40]. While these self-report measures were consistent across surveys, they were collected using different methods. Ausplay and the NSW PHS used CATI, whereas the Active Kids registration platform used an online bespoke government platform, which may have different effects on socially desirable responding. Another limitation of this study was the change in survey methods over time. The Ausplay survey changed from a dual frame (including landline and mobile phone users) to a mobile only frame in 2019. Similarly, the NSW PHS changed from a dual from to a single mobile frame in 2021. It is also important to note that the response rate in the NSW PHS decreased between 2017 and 2022. However, using survey weights in the analyses aims to overcome the design changes and reduced response rate.

## Conclusions

Using population-level data, we found no increases in physical activity or sport participation in children and adolescents in NSW between 2017 and 2022. However, it is important to interpret these findings with COVID-19 in mind and it is possible that the Active Kids program may have attenuated the impact of the COVID-19 community restrictions on sport participation in NSW. While previous work shows that children and adolescents who engaged in the Active Kids program reported an increase in their physical activity levels, these increases were not sufficient to influence overall physical activity and sport participation at a population level in NSW. Targeted focus on addressing the inequalities in physical activity and sport participation amongst children and adolescents must remain a priority.

## Electronic supplementary material

Below is the link to the electronic supplementary material.

Supplementary Material 1

Supplementary Material 2

## Data Availability

No datasets were generated or analysed during the current study.

## Abbreviations

CATI: Computer assisted telephone interviews
CFTC: Child Fitness Tax Credit
CI: Confidence intervals
MVPA: Moderate-to-vigorous physical activity
NSW: New South Wales
PHS: Population Health Survey
PPR: Proportional reporting ratio
SEIFA: Socio-Economic Indexes for Areas
