# Supplementary material for: Population level effects of the active kids program on children and adolescents’ physical activity and sport participation in NSW, Australia
Source: Int J Behav Nutr Phys Act. 2025 May 28;22:65. doi: 10.1186/s12966-025-01763-2 (PMC12117741; doi:10.1186/s12966-025-01763-2)
Supplement: Supplementary file 2 — Supplementary Material 2 [file 12966_2025_1763_MOESM2_ESM.docx]

Supplementary Table 1. Prevalence ratios for the proportion of children aged 5-18 meeting physical activity guidelines in NSW (PHS)

|  | 2017 (n=1655)  PR (95% CI) | 2018  (n=1657)  PR (95% CI) | 2019  (n=1467)  PR (95% CI) | 2020  (n=1617)  PR (95% CI) | 2021  (n=1490)  PR (95% CI) | 2022  (n=1445)  PR (95% CI) |
| --- | --- | --- | --- | --- | --- | --- |
| Age group |  |  |  |  |  |  |
| 5-8 years | Reference | Reference | Reference | Reference | Reference | Reference |
| 9-11 years | 0.89 (0.62,1.27) | 0.90 (0.65,1.26) | 0.86 (0.58,1.28) | 0.61 (0.37,1.00) | 0.97 (0.64,1.48) | 0.56 (0.37,0.86) |
| 12-14 years | 0.63 (0.43,0.92) | 0.66 (0.47,0.93) | 0.49 (0.33,0.73) | 0.56 (0.33,0.95) | 0.71 (0.45,1.13) | 0.50 (0.33,0.76) |
| 15-18 years | 1.99 (1.53,2.58) | 1.87 (1.45,2.40) | 1.93 (1.45,2.56) | 2.97 (2.12,4.15) | 2.19 (1.61,2.96) | 1.81 (1.37,2.39) |
| Gender |  |  |  |  |  |  |
| Males | Reference | Reference | Reference | Reference | Reference | Reference |
| Females | 0.69 (0.55,0.86) | 0.71 (0.58,0.88) | 0.86 (0.67,1.11) | 0.80 (0.59,1.09) | 0.90 (0.69,1.17) | 0.67 (0.51,0.87) |
| Socioeconomic status |  |  |  |  |  |  |
| 1st (most disadvantaged) | Reference | Reference | Reference | Reference | Reference | Reference |
| 2nd | 0.91 (0.65,1.28) | 0.98 (0.71,1.35) | 1.16 (0.79,1.70) | 1.02 (0.68,1.54) | 0.90 (0.61,1.34) | 1.31 (0.89,1.93) |
| 3rd | 1.06 (0.77,1.46) | 0.96 (0.69,1.32) | 1.23 (0.84,1.79) | 1.10 (0.74,1.65) | 0.99 (0.68,1.44) | 1.41 (0.96,2.07) |
| 4th | 1.25 (0.91,1.70) | 1.02 (0.75,1.39) | 1.18 (0.82,1.69) | 0.84 (0.53,1.32) | 1.11 (0.76,1.61) | 1.43 (0.95,2.16) |
| 5th (least disadvantaged) | 0.92 (0.61,1.37) | 1.23 (0.90,1.69) | 1.41 (0.94,2.11) | 0.89 (0.52,1.52) | 0.92 (0.56,1.52) | 1.13 (0.71,1.80) |

Note. in the NSW PHS different physical activity questions are asked and meeting guidelines is classified differently for the 5–15-year age group and the 16–18-year age group. For children aged 5-15, meeting physical activity guidelines is defined as at least 60 minutes of physical activity each day; for children aged 16-18, the physical activity guidelines are classified as at least 150 minutes of physical activity a week.

Supplementary Table 2. Prevalence ratios for the proportion of children aged 4-18 years participating in sport at least once per week in NSW (Ausplay)

|  | 2017 (n=1655)  PR (95% CI) | 2018  (n=1657)  PR (95% CI) | 2019  (n=1467)  PR (95% CI) | 2020  (n=1617)  PR (95% CI) | 2021  (n=1490)  PR (95% CI) | 2022  (n=1445)  PR (95% CI) |
| --- | --- | --- | --- | --- | --- | --- |
| Age group |  |  |  |  |  |  |
| 4-8 years | Reference | Reference | Reference | Reference | Reference | Reference |
| 9-11 years | 1.21 (1.12,1.30) | 1.36 (1.24,1.48) | 1.28 (1.18,1.39) | 1.44 (1.32,1.58) | 1.34 (1.21,1.48) | 1.26 (1.14,1.38) |
| 12-14 years | 1.19 (1.10,1.29) | 1.25 (1.14,1.37) | 1.19 (1.09,1.30) | 1.38 (1.26,1.51) | 1.28 (1.15,1.42) | 1.25 (1.15,1.36) |
| 15-18 years | 1.41 (1.31,1.51) | 1.54 (1.42,1.68) | 1.49 (1.39,1.60) | 1.84 (1.70,2.00) | 1.90 (1.74,2.08) | 1.64 (1.50,1.78) |
| Gender |  |  |  |  |  |  |
| Males | Reference | Reference | Reference | Reference | Reference | Reference |
| Females | 0.69 (0.55,0.86) | 0.71 (0.58,0.88) | 0.86 (0.67,1.11) | 0.80 (0.59,1.09) | 0.90 (0.69,1.17) | 0.67 (0.51,0.87) |
| Socioeconomic status |  |  |  |  |  |  |
| 1st (most disadvantaged) | Reference | Reference | Reference | Reference | Reference | Reference |
| 2nd | 1.09 (0.96,1.23) | 1.06 (0.92,1.21) | 0.96 (0.85,1.08) | 1.13 (1.00,1.28) | 1.03 (0.89,1.18) | 1.18 (1.03,1.35) |
| 3rd | 1.11 (0.99,1.25) | 1.12 (0.99,1.27) | 1.07 (0.96,1.20) | 1.16 (1.03,1.31) | 1.10 (0.97,1.26) | 1.25 (1.10,1.42) |
| 4th (least disadvantaged) | 1.24 (1.11,1.38) | 1.21 (1.07,1.36) | 1.19 (1.07,1.32) | 1.34 (1.20,1.49) | 1.20 (1.06,1.36) | 1.40 (1.24,1.58) |
